# Effect of planned place of birth on obstetric interventions and maternal outcomes among low-risk women: a cohort study in the Netherlands

**DOI:** 10.1186/s12884-016-1130-6

**Published:** 2016-10-28

**Authors:** N. Bolten, A. de Jonge, E. Zwagerman, P. Zwagerman, T. Klomp, J. J. Zwart, C. C. Geerts

**Affiliations:** 1 Department of Midwifery Science, AVAG and the EMGO Institute of Health and Care Research, VU University Medical Centre, Amsterdam, The Netherlands; 2 Midwife Academy Amsterdam, AVAG, Amsterdam, The Netherlands; 3 Department of Obstetrics and Gynaecology, Deventer Hospital, Deventer, The Netherlands

**Keywords:** Midwifery, Caesarean section, Instrumental birth, Obstetrical, Perineal damage, Postpartum haemorrhage, Home childbirth

## Abstract

**Background:**

The use of interventions in childbirth has increased the past decades. There is concern that some women might receive more interventions than they really need. For low-risk women, midwife-led birth settings may be of importance as a counterbalance towards the increasing rate of interventions. The effect of planned place of birth on interventions in the Netherlands is not yet clear. This study aims to give insight into differences in obstetric interventions and maternal outcomes for planned home versus planned hospital birth among women in midwife-led care.

**Methods:**

Women from twenty practices across the Netherlands were included in 2009 and 2010. Of these, 3495 were low-risk and in midwife-led care at the onset of labour. Information about planned place of birth and outcomes, including instrumental birth (caesarean section, vacuum or forceps birth), labour augmentation, episiotomy, oxytocin in third stage, postpartum haemorrhage >1000 ml and perineal damage, came from the national midwife-led care perinatal database, and a postpartum questionnaire.

**Results:**

Women who planned home birth more often had spontaneous birth (nulliparous women aOR 1.38, 95 % CI 1.08–1.76, parous women aOR 2.29, 95 % CI 1.21–4.36) and less often episiotomy (nulliparous women aOR 0.73, 0.58–0.91, parous women aOR 0.47, 0.33–0.68) and use of oxytocin in the third stage (nulliparous women aOR 0.58, 0.42–0.80, parous women aOR 0.47, 0.37–0.60) compared to women who planned hospital birth. Nulliparous women more often had anal sphincter damage (aOR 1.75, 1.01–3.03), but the difference was not statistically significant if women who had caesarean sections were excluded. Parous women less often had labour augmentation (aOR 0.55, 0.36–0.82) and more often an intact perineum (aOR 1.65, 1.34–2.03). There were no differences in rates of vacuum/forceps birth, unplanned caesarean section and postpartum haemorrhage >1000 ml.

**Conclusions:**

Women who planned home birth were more likely to give birth spontaneously and had fewer medical interventions.

## Background

During the past decades, the use of interventions in childbirth has increased, including caesarean sections. In the Netherlands the caesarean section rate has slowly increased from 14.8 % in 2003 [1] to 16.4 % in 2013 [2]. In other Western countries the increase has been much larger [3–6]. The World Health Organization (WHO) stated that at population level, caesarean section rates higher than 10 % are not associated with reductions in maternal and newborn mortality rates [7]. Consequently, there is major concern that some women might receive more interventions than they really need.

Unnecessary interventions should be avoided, because they may be associated with negative maternal health outcomes, such as postpartum haemorrhage (PPH) and increased health risks for future pregnancies [8–10].

Midwife-led birth settings have been associated with a lower rate of severe adverse maternal morbidity [11]. Additionally, international studies showed a significantly lower risk of episiotomy [12, 13], pharmacological pain management [12–15], assisted vaginal birth [12, 13], caesarean section [12, 13], and augmentation of labour [12–15] in birth settings other than obstetric units, although in one study no difference in rate of instrumental births was found [15].

However, these studies have been conducted in countries with relatively low home birth rates, not exceeding 7% [12, 13, 15]. In the Netherlands, home birth has traditionally been part of the established maternity care system. Although the home birth rate is falling, it is still higher than in other Western countries (about 20 %) [16]. There has been much debate about the safety of home births. However, a recent Dutch study among 743.070 low risk women showed similar rates of perinatal morbidity and mortality among planned home and planned midwife-led hospital births [17]. Another study showed no increased rates of severe maternal morbidity among planned home births [11]. Less is known about differences in interventions and other maternal outcomes.

Only few Dutch studies have examined the association between planned place of birth and interventions [18, 19]. One of these studies showed significantly fewer interventions among parous women who planned home birth between 1998 and 1999 [18], but another study did not confirm this [19]. So, these findings are not conclusive. Larger studies are needed into the relationship between planned place of birth and obstetric interventions in the Netherlands. In some other Western countries the rate of home births is increasing [20–22]. The findings of this study can be important for these countries, because we can provide a large enough dataset to show potential differences in outcomes between planned home and planned hospital births among low-risk women in the Netherlands.

Pregnant women who have a choice of place of birth need to receive evidence based information from their midwife to make a well-informed choice in place of birth. Our study aimed to examine the differences in rates of obstetric interventions and maternal outcomes between planned home birth and planned hospital birth among low-risk women in midwife-led care at the onset of labour.

## Methods

In the Netherlands, almost 170,000 births occurred in 2013 [2]. From 50.6 % of the women, the start of labour was in midwife-led care, the remaining 49,4 % started their labour in obstetrician-led care. In 2012, 2,692 practising midwives were registered, of which 72 % worked in a midwife-led care setting spread out over 519 practices across the country. The remaining 28 % worked in a obstetrician-led care setting, under the responsibility of obstetricians [23]. A pregnant woman is defined low-risk when she has good general health and an uncomplicated medical and obstetric history. Low-risk women have a free choice of place of birth: they can give birth at home or in hospital in obstetric units under responsibility of a midwife [24]. If risk factors arise during pregnancy, labour or in the postpartum period, the care of the women will be transferred from midwife-led to obstetrician-led care. Indications for transfer from midwife-led to obstetrician-led care are laid out in the ‘List of Obstetric Indications’ (VIL) [24]. This list is revised regularly by a project group consisting of midwives, obstetricians, paediatricians, and general practitioners. Reasons of transfer during labour can be, for example, the use of pharmacological pain relief, meconium stained fluid, preterm birth, failure to progress during first - or second stage of labour or signs of fetal distress. Obstetric interventions in obstetrician-led care studied are caesarean section, vacuum/forceps birth, labour augmentation or administration of pharmacological pain relief. Episiotomy is performed and repaired in both midwife-led and obstetrician-led care.

### Study design

This cohort study is part of the DELIVER (Data EersteLIjns VERloskunde) study. The study design of the DELIVER study is a prospective multicentre cohort study, and has been described in detail elsewhere [25]. In short, from twenty midwifery practices across the Netherlands, women were recruited to participate and the response rate was 62 %. Data were collected between September 2009 and March 2011. Clients filled in a questionnaire approximately 6 weeks postpartum. Data of these women were linked to national midwife-led care registration data: the National Perinatal Database-1 (NPD-1), and routine antenatal care data recorded by their midwives. Overall linkage was successful in 76.1 % of women. Most results in this study are derived from NPD-1, sometimes complemented with information from the questionnaire. For pharmacological pain relief, position during second stage of labour and BMI we used the questionnaire. For BMI we used the routine antenatal care data if it was missing on the questionnaire. Agreement between the questionnaire and the NPD-1 was generally high, for example 97.0 % for vacuum or forceps birth and 98.7 % for caesarean section. For BMI the interclass correlation coefficient between the routine antenatal data and the questionnaire was 0.95.

The design of the DELIVER study was approved by the Medical Ethics Committee of the VU University Medical Centre Amsterdam [25]. All participants were informed about the study and they were asked to participate by their consulting midwife. Informed consent was obtained verbally. Client participation was voluntary and they could withdraw at any time.

### Study population

For this study we used data from low-risk women who were in midwife-led care at the onset of labour. Women who were transferred to obstetrician-led care during pregnancy, and who received midwife-led care but had a ‘medium risk’ indication (according to VIL) [24], including a history of PPH or manual removal of the placenta, and who had prolonged rupture of membranes without contractions were excluded from our study population, because they were advised to give birth in hospital and thus did not have a choice in their place of birth.

### Definition of variables

The determinant is the intended place of birth, home or hospital, which is discussed with the midwife during pregnancy and recorded in the pregnancy card and in the NPD-1. Sometimes this is not recorded, mainly because the woman has not yet made a choice before labour.

Main outcomes were spontaneous birth (yes/no), obstetric interventions and maternal outcomes. Obstetric interventions included: vacuum/forceps birth (yes/no), unplanned caesarean section (yes/no), episiotomy (yes/no), labour augmentation using oxytocin (yes/no) and oxytocin during third stage of labour (yes/no).

The following maternal outcomes were studied: intact perineum (stitches, yes/no), anal sphincter damage (third or fourth degree tear, yes/no) and estimated blood loss >1000 ml (yes/no).

Secondary outcomes were maternal position at the time of birth (recumbent or non-recumbent), recumbent was defined as women who gave birth while in a lying position, non-recumbent included all other positions, use of pharmacological pain management (yes/no), transfer to obstetrician-led care during labour (during first, second or third stage) and duration of first stage (<6 h or >12 h, based on categories registered in NPD-1) and second stage (>30 min for parous women or >90 min for nulliparous women) [26, 27].

Pharmacological pain relief included the use of intramuscular or intravenous opioids and/or epidural analgesia.

If a woman is already in hospital for a planned hospital birth the responsible caregiver changes and the woman will give birth in the room where she was. In some cases, she is transported to another room within the same hospital. When a woman has to be transferred from home, she will travel by car or, if necessary, by ambulance.

### Potential confounders

We adjusted the results for the following factors that are known to be potential confounders in the relationship between planned place of birth and obstetric interventions or maternal outcomes: maternal age [28–31] categorised as < 25 year, 25–35 year and ≥35 year; gestational age [32] categorised as 37–37 + 6, 38–40 + 6, 41–41 + 6 weeks; body mass index [33, 34] categorized as < 25 and ≥ 25 kg/m^2^; ethnic background [35–37] and socioeconomic position [29, 33, 38]. Ethnic background was categorised as Dutch (both parents of the pregnant women born in the Netherlands), non-Dutch Western background (at least one parent born in another country in Europe except for Turkey, or born in Oceania, Indonesia, North-America or Japan) or non-Western background (at least one parent born in Africa, Latin-America, Asia or Turkey) [38]. The categorisation is based on the definition of Statistics Netherlands [39]. Only three groups could be created due to the size of the study-population. Socioeconomic position was categorised as: high, medium and low. This categorization is determined by the first four digits of the woman’s postal code and is based on three elements: the mean household income level of the neighbourhood, employment and highest education [39].

### Data analysis

The analysis was stratified by parity, because it is known that the magnitude of the effect of planned place of birth on obstetric interventions, maternal outcomes and labour processes varies for nulliparous and parous women [18, 19, 40, 41].

We used frequencies to describe baseline characteristics for women who planned home or hospital birth. Possible differences were tested with the chi-square test.

The associations between planned place of birth and, separately, spontaneous birth, obstetric interventions, maternal outcomes and labour process, were analysed using univariable logistic regression. Multivariable logistic regressions were used to adjust the associations for confounders.

To account for clustering of women within midwifery practices, multilevel analyses were performed.

We used data from women who started labour in midwife-led care. For most women start of labour in midwife-led or obstetrician-led care was clear, but information of the NPD-1 showed discrepancies for the onset of labour. For example in some cases it was registered that a woman was transferred during pregnancy, but the reason for transfer indicated that the women was already in labour (e.g. meconium stained fluid). We conducted sensitivity analyses for maternal outcomes and interventions including women with and without discrepancies in the definition for start of labour in midwife-led care.

The current study was too small to analyse the difference between planned place of birth and perinatal mortality or morbidity. Frequency of perinatal mortality and Apgar score < 7 after 5 min was reported for planned place of birth.

Results were reported as adjusted odds ratios (aOR) and 95 % confidence intervals (CI). All analyses were performed using SPSS version 20.0 and STATA version 12. Statistical significance was defined as p-value <0.05.

## Results

### Participants

**Fig. 1:**
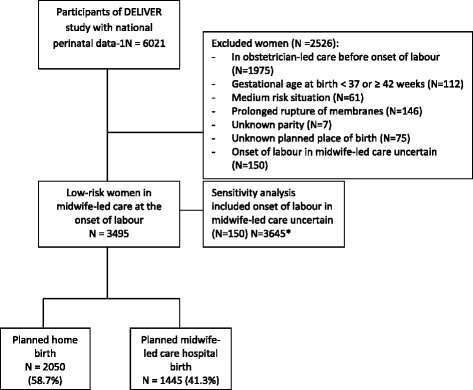
Flow diagram of women in the study. *For sensitivity analysis, women with start childbirth in midwife-led care uncertain were included (*N* = 150). **Questionnaire data was used for pharmacological pain relief and position during childbirth. Data from questionnaire was available for *N* = 2160

In the Deliver study, NPD-1 were available and linked to the questionnaire data from 6021 (76.1 %) women. Of these 3495 (58.0 %) women were low-risk at the onset of labour, 2050 (58.7 %) of whom planned home birth and 1445 (41.3 %) planned hospital birth (See Fig. 1). Data from the questionnaire was available for 2160 women (61.8 %).

Table 1 shows the baseline characteristics. Compared to women who planned hospital birth, nulliparous women who planned home birth were more often between 25 and 35 years old, there was no significant difference in age for parous women. Women who planned home birth more often had a Dutch background. Parous women with a history of instrumental vaginal birth more often planned a hospital birth.

**Table 1:** Characteristics of women who planned home birth and women who planned hospital birth (*N* = 3495)

	Planned place of birth					
	Nulliparous women (*N* = 1585)			Parous women (*N* = 1910)		
	Home birth (*N* = 868) N(%)	Hospital birth (*N* = 717) N(%)	*P*-value	Home birth (*N* = 1182) N(%)	Hospital birth (*N* = 728) N(%)	*P*-value
Maternal age, years						
< 25	145 (16.7)	131 (18.3)	0.002*	48 (4.1)	36 (4.9)	0.148
25–35	651 (75.0)	490 (68.3)		870 (73.6)	506 (69.5)	
≥ 35	72 (8.3)	96 (13.4)		264 (22.3)	186 (25.5)	
Gestational age at birth, weeks						
37 + 0–37 + 6	35 (4.0)	33 (4.6)	0.201	27 (2.3)	14 (1.9)	0.856
38 + 0–40 + 6	641(73.8)	551 (76.8)		934 (79.0)	575 (79.0)	
41 + 0–41 + 6	192 (22.1)	133 (18.5)		221 (18.7)	139 (19.1)	
Ethnic background						
Dutch background	785 (90.4)	540 (75.3)	<0.001*	1030 (87.1)	532 (73.1)	<0.001*
Non-Dutch Western background	37 (4.3)	82 (11.4)		82 (6.9)	69 (9.5)	
Non-Western background	43 (5.0)	92 (12.8)		65 (5.5)	125 (17.2)	
Missing	3 (0.3)	3 (0.4)		5 (0.4)	2 (0.3)	
Prepregnancy BMI						
< 25.0	654 (75.3)	511 (71.3)	0.146	842 (71.2)	481 (66.1)	0.051
≥ 25.0	182 (21.0)	170 (23.7)		292 (24.7)	216 (29.7)	
Missing	32 (3.7)	36 (5.0)		48 (4.1)	31 (4.3)	
Socioeconomic position						
High	187 (21.5)	180 (25.1)	0.181	321 (27.2)	205 (28.2)	0.137
Middle	389 (44.8)	319 (44.5)		581 (49.2)	325 (44.6)	
Low	288 (33.2)	215 (30.0)		277 (23.4)	194 (26.6)	
Missing	4 (0.5)	3 (0.4)		3 (0.3)	4 (0.5)	
Birth weight						
< 2500	8 (0.9)	9 (1.3)	0.553	3 (0.3)	0	0.053
2500–2999	88 (10.1)	79 (11.0)		49 (4.1)	51 (7.0)	
3000–3499	351 (40.4)	312 (43.5)		373 (31.6)	220 (30.2)	
3500–3999	311 (35.8)	238 (33.2)		471 (39.8)	287 (39.4)	
≥ 4000	104 (12.0)	76 (10.6)		286 (24.2)	169 (23.2)	
Missing	6 (0.7)	3 (0.4)		0	1 (0.1)	
Instrumental vaginal birth in obstetric history						
Yes				98 (8.3)	116 (15.9)	<0,001*

Data shown: no. (%) of women

*p < 0.05

### Obstetric interventions and maternal outcomes

Table 2 shows that women who planned home birth more often had a spontaneous birth compared to women who planned hospital birth (nulliparous women aOR 1.38, 95 % CI 1.08–1.76 and parous women aOR 2.29, 95 % CI 1.21–4.36). Women who planned home birth had fewer episiotomies (nulliparous women aOR 0.73, 95 % CI 0.58–0.91 and parous women aOR 0.47, 95 % CI 0.33–0.68) and less frequently use of oxytocin in the third stage of labour (nulliparous women aOR 0.58, 95 % CI 0.42–0.80 and parous women aOR 0.47, 95 % CI 0.37–0.60) compared to women who planned hospital birth. Parous women who planned birth at home less often required oxytocin for augmentation (aOR 0.55, 95 % CI 0.36–0.82). Maternal outcomes showed that parous women who planned birth at home were more likely to have an intact perineum (aOR 1.65, 95 % CI 1.34–2.03) compared to those who planned hospital birth. Nulliparous women who planned home birth more often had anal sphincter damage (aOR 1.75, 95 % CI 1.01–3.03). When we restricted our analysis to nulliparous women who did not have a caesarean section, because these women cannot have perineal damage, the association became non-significant: aOR 1.71, 95 % CI 0.98–2.97 (not in table). There were no differences in vacuum-forceps birth, unplanned caesarean section and the rate of blood loss >1000 ml.

**Table 2:** Obstetric interventions and maternal outcomes, planned home and hospital birth in low-risk women

	Nulliparous (*n* = 1585)				Parous (*n* = 1910)			
	No of events/births	Incidence (%)	Crude OR (95 % CI)	Adjusted OR (95 % CI)^a^	No of events/births	Incidence (%)	Crude OR (95 % CI)	Adjusted OR (95 % CI)^a^
Spontaneous birth								
Planned home birth	651/868	75.0	1.31 (1.05–1.65)*	1.38 (1.08–1.76)*	1164/1182	98.5	2.30 (1.25–4.25)*	2.29 (1.21–4.36)*
Planned hospital birth	498/717	69.5	1	1	703/728	96.6	1	1
Vacuum/forceps birth ^c^								
Planned home birth	156/868	18.0	0.83 (0.64–1.06)	0.77 (0.60–1.01)	11/1182	0.9	0.52 (0.23–1.16)	0.46 (0.20–1.07)^b^
Planned hospital birth	150/717	20.9	1	1	13/728	1.8	1	1
Unplanned caesarean section								
Planned home birth	61/868	7.0	0.74 (0.51–1.07)	0.72 (0.48–1.09)	7/1182	0.6	0.35 (0.14–0.91)*	0.42 (0.16–1.10)^b^
Planned hospital birth	69/717	9.6	1	1	12/728	1.7	1	1
Episiotomy								
Planned home birth	319/856	37.3	0.81 (0.65–1.00)	0.73 (0.58–0.91)*	74/1182	6.3	0.49 (0.35–0.69)*	0.47 (0.33–0.68)*
Planned hospital birth	299/713	41.9	1	1	89/727	12.2	1	1
Labour augmentation: oxytocin								
Planned home birth	208/868	24.0	0.77 (0.60–0.99)*	0.81 (0.62–1.05)	50/1182	4.2	0.49 (0.33–0.73)*	0.55 (0.36–0.82)*
Planned hospital birth	202/717	28.2	1	1	60/728	8.2	1	1
Use of oxytocin in the third stage of labour								
Planned home birth	694/857	81.0	0.62 (0.45–0.84)*	0.58 (0.42–0.80)*	739/1181	62.6	0.45 (0.36–0.57)*	0.47 (0.37–0.60)*
Planned hospital birth	616/713	86.4	1	1	549/727	75.5	1	1
Maternal outcomes								
Intact perineum								
Planned home birth	220/856	25.7	0.87 (0.69–1.11)	0.91 (0.71–1.18)	575/1182	48.7	1.55 (1.28–1.89)*	1.65 (1.34–2.03)*
Planned hospital birth	210/713	29.5	1	1	285/727	39.2	1	1
Anal sphincter damage (third-or fourth degree)								
Planned home birth	45/856	5.3	1.67 (0.99–2.81)	1.75 (1.01–3.03)*^b^	17/1182	1.4	0.74 (0.36–1.52)	0.73 (0.34–1.58)^b^
Planned hospital birth	23/713	3.2	1	1	14/727	1.9	1	1
Haemorrhage postpartum >1000 ml								
Planned home birth	65/847	7.7	1.21 (0.81–1.82)	1.03 (0.67–1.59)	28/1180	2.4	0.67 (0.38–1.19)	0.68 (0.38–1.23)^b^
Planned hospital birth	46/708	6.5	1	1	24/725	3.3	1	1

Multilevel analysis of obstetric interventions and maternal outcomes

*p < 0.05

^a^Adjusted for maternal age, gestational age, ethnic background (Dutch/western background/non-western background), Body Mass Index (BMI), socio-economic position

^b^Adjusted for maternal age, ethnic background (Dutch/non-Dutch) and BMI if there were less than 90 cases to take account to the rule of ten events per variable

^c^If caesarean section after failed vacuum or forceps, this was analyzed as caesarean section, *N* = 18

Among the 3495 low - risk women there were 3 perinatal deaths; 1 in the planned home birth group and 2 in the planned hospital birth group. The incidence of Apgar score < 7 after 5 min was 11/2043 (0,5 %) in the planned home birth group, and 10/1443 (0,7 %) in the planned hospital birth group.

### Labour process

Table 3 shows that parous women who planned home birth more often had a duration of the first stage of labour less than six hours (aOR 1.74, 95 % CI 1.38–2.19) and they less often had a longer duration of the second stage (aOR 0.65, 95 % CI 0.47–0.91). Nulliparous women who planned home birth had more often a longer duration of the second stage (aOR 1.38, 95 % CI 1.03–1.86). Nulliparous and parous women who planned home birth were more likely to give birth in non-recumbent position (nulliparous women aOR 1.98, 95 % CI 1.27–3.10 and parous women aOR 1.56, 95 % CI 1.08–2.25) and they used less pharmacological pain relief (nulliparous women aOR 0.53, 95 % CI 0.39–0.72 and parous women aOR 0.15, 95 % CI 0.07–0.30). Women who planned home birth were less often transferred to obstetrician-led care during labour or directly postpartum (nulliparous women aOR 0.57, 95 % CI 0.45–0.72 and parous women aOR 0.39, 95 % CI 0.31–0.49). Fewer women in the planned home birth group were transferred during the first stage of labour (nulliparous women aOR 0.54, 95 % CI 0.43–0.68 and parous women aOR 0.32, 95 % CI 0.24–0.42). We found no differences in transfer of care in second and third stage of labour among parous woman. Nulliparous women who planned home birth were more often transferred during third stage of labour (aOR 1.70, 95 % CI 1.05–2.77). There was no difference in transfer-rate among nulliparous women during second stage of labour. When we restricted the analysis to women who were not transferred earlier during labour (e.g. not in first or second stage) the association between planned place of birth and transfer during third stage among nulliparous women became non-significant (aOR 1.22, 95 % CI 0.73–2.03).

**Table 3:** Labour process among women who started labour in midwife-led care, planned home versus hospital birth

	Nulliparous (*n* = 1585)				Parous (*n* = 1910)			
	No of events/Births	Incidence (%)	Crude OR (95 % CI)	Adjusted OR (95 % CI) ^a^	No of events/births	Incidence (%)	Crude OR (95 % CI)	Adjusted OR (95 % CI) ^a^
Duration first stage: < 6 h								
Planned home birth	213/825	25.8	0.99 (0.78–1.27)	0.99 (0.76–1.28)	944/1173	80.5	1.89 (1.52–2.35)*	1.74 (1.38–2.19)*
Planned hospital birth	173/693	25.0	1	1	496/724	68.5	1	1
Duration first stage: > 12 h								
Planned home birth	213/825	25.8	0.85 (0.68–1.08)	0.86 (0.67–1.11)	26/1173	2.2	0.64 (0.36–1.12)	0.69 (0.38–1.25)^b^
Planned hospital birth	204/693	29.4	1	1	26/724	3.6	1	1
Duration second stage nulliparous women: ≥ 90 min								
Planned home birth	179/803	21.2	1.57 (1.18–2.09)*	1.38 (1.03–1.1.86)*				
Planned hospital birth	94/661	14.2	1	1				
Duration second stage parous women: ≥ 30 min								
Planned home birth					95/1175	8.1	0.69 (0.50–0.95)*	0.65 (0.47–0.91)*
Planned hospital birth					83/715	11.6	1	1
Position during childbirth: non- recumbent^c^								
Planned home birth	98/579	16.9	1.85 (1.22–2.80)*	1.98 (1.27–3.10)*	138/792	17.7	1.47 (1.03–2.09)*	1.56 (1.08–2.25)*
Planned hospital birth	45/415	10,8	1	1	55/417	13.4	1	1
Use of pharmacological pain relief^c/d^								
Planned home birth	139/584	23.8	0.54 (0.40–72)*	0.53 (0.39–0.72)*	11/800	1.4	0.13 (0.07–0.26)*	0.15 (0.07–0.30)*^b^
Planned hospital birth	151/418	36.1	1	1	40/421	9.5	1	1
Transfer of care to obstetrician during labour or directly postpartum								
Planned home birth	509/868	58.6	0.60 (0.48–0.74)*	0.57 (0.45–0.72)*	173/1182	14.6	0.36 (0.28–0.45)*	0.39 (0.31–0.49)*
Planned hospital birth	499/717	69.6	1	1	236/728	32.4	1	1
Transfer of care to obstetrician during first stage of labour								
Planned home birth	310/868	35.7	0.51 (0.41–0.63)*	0.54 (0.43–0.68)*	110/1182	9.3	0.29 (0.23–0.38)*	0.32 (0.24–0.42)*
Planned hospital birth	365/717	50.9	1	1	188/728	25.8	1	1
Transfer of care to obstetrician during second stage of labour								
Planned home birth	141/868	16.2	1.11 (0.84–1.45)	0.94 (0.70–1.25)	17/1182	1.4	0.62 (0.31–1.23)	0.63 (0.31–1.30)^b^
Planned hospital birth	107/717	14.9	1	1	17/728	2.3	1	1
Transfer of care to obstetrician directly postpartum								
Planned home birth	58/868	6.7	1.62 (1.02–2.58)*	1.70 (1.05–2.77)*^b^	46/1182	3.9	0.90 (0.56–1.45)	0.96 (0.58–1.57)^b^
Planned hospital birth	27/717	3.8	1	1	31/728	4.3	1	1

Multilevel analysis of obstetric interventions and maternal outcomes

*p < 0.05

^a^Adjusted for maternal age, gestational age, ethnic background (Dutch/western background/non-western background), Body Mass Index (BMI), socio-economic position

^b^Adjusted for maternal age, ethnic background (Dutch/non-Dutch) and BMI if there were less than 90 cases, to take account to the rule of ten events per variable

^c^Information extracted from the third (postpartum) questionnaire (available for *N* = 2160 women)

^d^Nulliparous women with pharmacological pain relief used intramuscular opioids in 24,1 %, intravenous opioids in 24,5 %, epidural analgesia in 62,8 % or other 4,1 %

Parous women with pharmacological pain relief used intramuscular opioids in 37,3 %, intravenous opioids in 33,3 % and epidural analgesia in 27,5 % or other 11,8 %. Numbers do not count to hundred percent because some women received more than one form of medical pain medication

### Sensitivity analysis

After adding 150 women who probably started labour in midwife-led care, although there were some inconsistencies in their data, results for most maternal outcomes and interventions were similar. For one outcome, the statistical significance changed. The difference in anal sphincter damage among nulliparous women who planned home birth compared to hospital birth became non-significant (aOR 1.57, 95 % CI 0.93–2.65).

## Discussion

### Main findings

This study shows that low-risk women in midwife-led care at the onset of labour who planned home birth were more likely to have a spontaneous birth, less likely to have an episiotomy, oxytocin in third stage and pharmacological pain relief and they less often were transferred during first stage of labour compared to those who planned hospital birth. There were no differences in vacuum/forceps birth, unplanned caesarean section and PPH >1000 ml.

Nulliparous women who planned home birth had a higher rate of anal sphincter damage, which was non-significant in the sensitivity analysis, including women of whom it was less certain whether they started labour in midwife-led care. They more often had a longer duration of the second stage of labour and they were more often transferred during third stage of labour.

Parous women who planned home birth were less likely to have labour augmentation, they more often had an intact perineum and a shorter first- and second stage of labour.

### Strengths and Limitations

A strength of this study is that we used a large prospective cohort study with a recent, diverse population of low-risk women. Although a randomized controlled trial would be the optimal design for studies into home birth and outcomes, this appeared not feasible, because women are not willing to be randomised for place of birth [42]. Without randomisation there could be differences between the study groups that cannot be measured or accounted for. However, in this observational study we controlled the associations for confounders, including BMI, to deal with unequally disturbed characteristics as much as possible. Furthermore, we accounted for clustering of women within midwifery practices.

For this study, data were used from NPD-1 and self-reported questionnaires. We showed good agreement between both sources, which suggests that data are of good quality.

A limitation of this study is that the population of the DELIVER study was higher educated and more often of Dutch ethnic background than the general Dutch population [25]. There is, however, no reason to assume that results would be in the opposite direction among women not included in this study.

### Interpretation

We found a higher rate of spontaneous birth, in agreement with other studies [12, 13, 43]. It is known that women who planned home birth might be more motivated to avoid interventions and have a more critical attitude towards labour technology [18]. In addition, parous women with a complicated previous birth (like instrumental birth) were more likely to opt for hospital birth in midwife-led care [19]. Their obstetric history may put them at higher risk of complications in the following pregnancy and thus they are less likely to give birth spontaneously [19]. Parous women with an uncomplicated birth in history more often prefer home birth [29]. In contrast to the Birthplace study, we found no statistically significant difference in caesarean section rate between planned birth settings [13]. An explanation for this can be that obstetricians in the Netherlands are reticent with caesarean section compared to other countries (WHO 2010: the Netherlands 15.6 %, the UK 23.8 %, Italy 38.8 %) [44]. Women who planned hospital birth in the Netherlands possibly have a lower risk for caesarean section than in the UK where women in an obstetric unit give birth under responsibility of the obstetrician. Other Dutch studies did show a lower risk for unplanned caesarean section for parous women who planned home birth, but they did not take confounders into account [18, 19].

Our study showed that women who planned home birth used pharmacological pain relief less frequently and parous women less often needed augmentation of labour [37]. Both augmentation and pain relief (particularly epidural analgesia) are associated with instrumental birth and therefore it seems likely that the lower risk for augmentation or pain relief in the planned home birth group might have contributed to more spontaneous births. Pain relief itself has been associated with augmentation, a longer duration of labour and more assisted vaginal deliveries [45]. The increased use of pharmacological pain relief may also explain the higher rate of transfer of care in the planned hospital birth group, since this cannot be administered in midwife-led care [37]. After transfer a woman and her baby will be monitored continuously. During monitoring it is more likely that concerns arise about the fetal condition, leading to more interventions [46]. During second stage of labour women who planned home birth more often were in non-recumbent position. Systematic reviews showed that giving birth in non-recumbent position is associated with fewer assisted deliveries, episiotomies and shorter duration of the second stage of labour [47, 48]. This might explain why the duration of second stage of labour was shorter for parous women and that there were fewer assisted deliveries and episiotomies for women who planned home birth in our study. On the other hand, nulliparous women who planned home birth more frequently had a longer duration of the second stage. A possible explanation could be that nulliparous women who plan home birth are less likely to be transferred to obstetric-led care, and thus more women stay low-risk throughout labour, allowing for a longer duration of second stage. Our hypothesis is that labourtime in midwife-led care during the second stage is significantly longer compared to the duration in hospital-care. Another explanation could be that the duration of second stage was increased due the transport-time in the portion of women who were transferred from home to hospital during second stage. Differences in labour duration should be interpreted with caution, because assessment of length of labour is very arbitrary.

Our results showed more frequent use of oxytocin in the third stage of labour among planned hospital births, but there was no difference in PPH >1000 ml. Our findings were not consistent with a recent Dutch study among 146,752 low-risk women, which showed significant less PPH in parous women who planned a home birth [40]. An international study too showed lower rates of PPH >1000 ml among births planned outside obstetric units for multiparous women [12]. However, these studies did not adjust the results for BMI, whereas we did. Increased BMI has been associated with a higher risk of postpartum haemorrhage for nulliparous women [49] and women with high BMI were more likely to give birth in hospital in our study.

Our study showed higher rates of anal sphincter tear among nulliparous women with a planned home versus planned hospital birth, although this association was not significant in the sensitivity analysis. Furthermore, it was not significant if women with caesarean sections were excluded. This suggests that some women who planned home birth and had anal sphincter damage might have had a caesarean section if they had planned hospital birth. Nevertheless, this finding is not in agreement with other international studies, showing that women who planned home birth had lower or similar rates of anal sphincter damage [12, 13, 43]. It is unclear why these studies found opposite results. One reason may be that women will be transferred from home to hospital if anal sphincter damage is suspected after birth. Even if this diagnosis is not confirmed in obstetrician-led care, anal sphincter damage may be registered in the midwife-led care registration as reason for transfer, and consequently these women might be classified as such in the dataset. In addition to a higher risk of anal sphincter damage in nulliparous women, we found a lower rate of episiotomy among nulliparous women who planned home birth [50]. The literature is inconclusive about the association between episiotomy and anal sphincter rupture [51, 52]. However, restrictive use of episiotomy has been recommended, as this is associated with less severe perineal damage [53]. Likewise, we found more intact perinea among parous women who planned home birth, which can be partly explained by the lower rate of episiotomies. Additional research is needed into the risk factors for perineal damage among nulliparous women who planned home birth.

The transfer rate to obstetrician-led care in third stage of labour was higher among nulliparous women in the planned home birth group. However, more women who planned home birth were at risk of being transferred during third stage, since a lower proportion of women was transferred during the first stage of labour compared to women who planned hospital birth.

However the chance of transfer to obstetrician-led care during labour or directly postpartum was lower for women with a planned home birth, although the rates are still relatively high especially for nulliparous women. This is of concern. A systematic review that compared the intrapartum transfer rates from home to hospital showed that in the Netherlands we have one of the highest numbers of intrapartum transfers [54]. In the Netherlands, women in midwife-led care are at very low risk of complications. When any medical intervention is indicated, such as continuous fetal monitoring or medical pain relief, transfer to obstetrician-led care will take place. There are initiatives to expand the scope of practice for primary care midwives, so that they can continue to care for women with moderate risk factors [55]. It is likely that this will reduce the transfer rate. A recent Dutch study shows a wide variation between transfer rates of midwifery practices [56].

## Conclusions

In conclusion, our study shows that low-risk women in midwife-led care at the onset of labour who planned home birth were more likely to have a spontaneous birth and a lower risk of obstetric interventions compared to women who planned hospital birth. The maternal outcomes showed more intact perinea in parous women and more anal sphincter damage in nulliparous women who planned home birth. The latter difference was no longer significant if caesarean sections were excluded. More research is needed into the risk factors for perineal damage in nulliparous women who plan home birth.
